# The SES-CD Could Be a Predictor of Short- and Long-Term Mucosal Healing After Exclusive Enteral Nutrition in Pediatric Crohn’s Disease Patients

**DOI:** 10.3389/fped.2022.874425

**Published:** 2022-05-18

**Authors:** Wenjuan Tang, Wenhui Hu, Peng Shi, Ziqing Ye, Jie Wu, Ye Zhang, Yuhuan Wang, Ying Huang

**Affiliations:** ^1^Department of Gastroenterology, Pediatric Inflammatory Bowel Disease Research Center, National Children’s Medical Center, Children’s Hospital of Fudan University, Shanghai, China; ^2^Medical Statistics Department, National Children’s Medical Center, Children’s Hospital of Fudan University, Shanghai, China

**Keywords:** simple endoscopic score for Crohn’s disease (SES-CD), exclusive enteral nutrition (EEN), mucosal healing (MH), Crohn’s disease, children

## Abstract

**Aims:**

To explore the predictors of mucosal healing (MH) for short- and long-term after exclusive enteral nutrition (EEN) in pediatric Crohn’s disease (CD) patients.

**Methods:**

A retrospective analysis was performed for newly diagnosed active CD patients admitted to our center from January 2017 to 30 December 2020, who were treated with EEN for induction therapy with a minimum of 12 months of follow-up post-EEN. According to the simple endoscopic score for CD (SES-CD), at 1-year post-EEN, 17 patients with an SES-CD < 3 were classified into the sustained MH group (sMH), and 33 patients with an SES-CD ≥ 3 were classified into the sustained non-MH group (sNMH). Statistical methods were used to compare the differences between the two groups and explore the predictors of MH at the end of EEN and 1-year post-EEN.

**Results:**

The SES-CD in the sMH group was lower than that in the sNMH group both at baseline and the end of EEN [sMH vs. sNMH: 8.7 ± 1.2 vs. 16.2 ± 1.0, respectively, *p* < 0.001 at baseline; 1.0 (3.5) vs. 4.0 (2.0), respectively, *p* < 0.01 at the end of EEN]. The weighted Pediatric Crohn’s Disease Activity Index and erythrocyte sedimentation rate in the sMH group were lower than those in the sNMH group at baseline (both *p* < 0.05), but showed no difference at the end of EEN. From baseline to 1-year post-EEN, compared with patients in the sNMH group, there were more patients classified with L1 in the sMH group at each time point (all *p* < 0.001) and fewer patients classified with L3 in the sMH group at baseline and 1-year post-EEN. After EEN, fewer patients received infliximab and had a longer exposure time to infliximab in the sMH group than in the sNMH group. Only the SES-CD at baseline was negatively associated with MH at the end of EEN (OR = 1.40 95% CI = 1.12–1.67, *p* = 0.00) and 1-year post-EEN (OR = 1.33, 95% CI = 1.12–1.58, *p* = 0.001), and the cut off value was 11.5.

**Conclusion:**

The SES-CD could predict both short- and long-term MH for EEN. Patients with an SES-CD < 11.5 had a high probability of reaching MH by EEN-inducing therapy and maintaining sustained MH at 1-year post-EEN. Patients with an SES-CD greater than 11.5 at baseline should be treated more aggressively with biologics.

## Introduction

Exclusive enteral nutrition (EEN) has been widely studied and recommended as a first-line therapy for inducing remission of Crohn’s disease (CD) in children (1, 2). Most studies reported that EEN effectively induced clinical and biochemical remissions and the endoscopic response of pediatric CD (3–6).

Mucosal healing (MH) is a treatment target for CD patients. The 2020 European Crohn’s and Colitis Organization-Pediatric IBD Porto group of the European Society of Pediatric Gastroenterology, Hepatology and Nutrition guideline defined MH as the absence of macroscopic inflammation or a simple endoscopic score for CD (SES-CD) < 3 points (7). For CD patients, clinical remission is not accurate in MH (8, 9), and maintaining sustained MH is associated with favorable long-term outcomes of CD progression (10). However, there have been limited studies on EEN using MH as the primary outcome in children (11–15), and the long-term effectiveness of EEN on MH is poorly documented.

Crohn’s disease is characterized by periods of remission and relapse, and the anti-inflammatory effect of EEN on the gut of CD patients is rapidly lost after food reintroduction (16, 17). Therefore, creating appropriate EEN programs and maintenance treatment options post-EEN to maintain sustained MH is important for patients. It is better to have some predictive variables of MH after EEN. A study demonstrated that a weighted Pediatric Crohn’s Disease Activity Index (wPCDAI) < 57, a fecal calprotectin (FCP) level < 500 ug/g, ileal involvement, and C-reactive protein level > 15 mg/L could be predictive factors of the clinical response to EEN (18). However, the clinical response did not correspond to MH in the gut.

Therefore, we designed this study. First, we identified the characteristics and clinical differences between patients with sustained MH and those with sustained non-MH at 1-year post-EEN. Second, we explored the predictive factors of MH for EEN at the end of EEN (short-term) and 1-year post-EEN (long-term).

## Materials and Methods

### Participants and Study Design

A retrospective analysis was performed for newly diagnosed active CD patients admitted to our center from January 2017 to 30 December 2020, who were treated with EEN for induction therapy with a minimum of 12 months of follow-up post-EEN. CD was diagnosed according to the Porto criteria (19) and based on a combination of history, physical and laboratory examinations, endoscopy with histology, and small bowel imaging (capsule endoscopy or magnetic resonance imaging or enhanced computerized tomography).

The feeding protocol was as follows: patients were exclusively treated with EEN for at least 6 weeks with no other medication. The formula could be polymeric or oligomeric, and the volume was determined according to the patient’s energy needs. The patients received high-energy intakes (>110–120% of the average requirement). For children ≤ 6 years old, the energy intakes were calculated according to weight; for children > 6 years old, the energy intakes were calculated according to age and sex (Supplementary Table 1). No other foods were permitted during EEN treatment, although the consumption of small amounts of water (<100 ml/day) was permitted. The feeding could be administered orally or **t**hrough a nasogastric tube.

The exclusion criteria were as follows: (1) patients who had genetic mutations or those with CD combined with another enteropathy, such as an Epstein–Barr virus or a tuberculosis infection; (2) patients who did not finish the daily prescribed volume of formula for any reason; (3) patients who had been given corticosteroids, immunosuppressive drugs, or biological agents before or during the EEN therapy process; and (4) patients with missing baseline, end of EEN, or 1-year post-EEN endoscopic imaging data.

**FIGURE 1:**
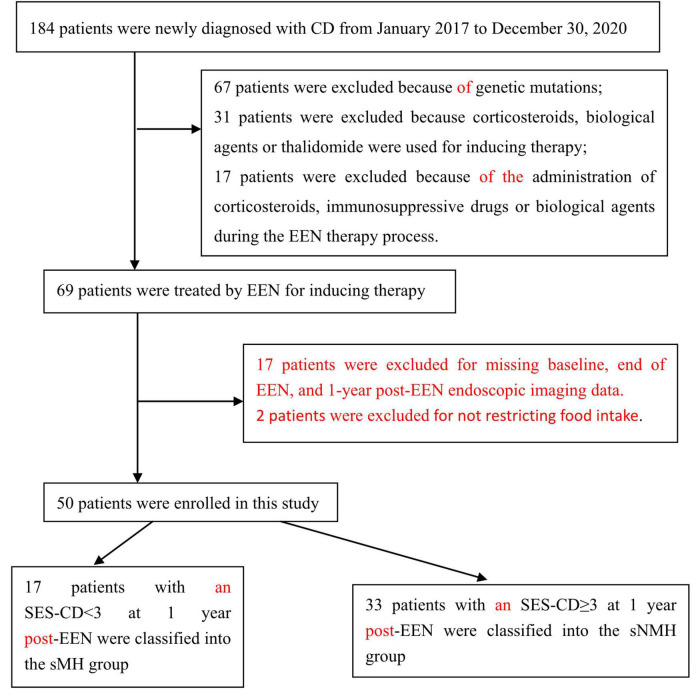
Flow diagram of patient inclusion in this study.

A total of 184 active CD patients were newly diagnosed from 2017.01 to 2020.12.31 (Figure 1); 67 patients were excluded because of genetic mutations, 31 patients were excluded because corticosteroids, biological agents, or thalidomide were used for inducing therapy, and 17 patients were excluded because corticosteroids, immunosuppressive drugs, or biological agents were used during the EEN therapy process. Hence, 69 patients were treated by EEN to induce the remission of CD. During the process, 17 patients were excluded for missing baseline, end of EEN, and 1-year post-EEN endoscopic imaging data. Two patients were excluded for not restricting their food intake. Hence, 50 patients were enrolled in this study. According to the SES-CD at 1-year post-EEN treatment, 17 patients with an SES-CD < 3 were classified into the sustained MH (sMH) group, and 33 patients with an SES-CD ≥ 3 were classified into the sustained non-MH (sNMH) group (Figure 1).

The medical records of these 50 patients from baseline (within 1 week before the start of EEN) to 2021.12.31 (at least 12 months of follow-up post-EEN) were extracted from existing hospital databases, including patient characteristics, disease activity data such as the SES-CD, Paris Classifications, Lewis score, wPCDAI, FCP, and erythrocyte sedimentation rate (ESR), nutrient data such as weight, height, serum albumin (ALB) level, hemoglobin (Hgb) level, and maintenance treatment post-EEN.

### Statistical Analysis

Data were analyzed using SPSS 26.0 (IBM, Armonk, NY, United States) and GraphPad Prism Software (GraphPad Software, San Diego, CA, United States). Categorical variables are expressed as numbers and proportions (%), and Fisher’s exact test was used to analyze the differences. Normally distributed quantitative variables are expressed as the means ± SD, and Student’s *t*-test was used to analyze the differences. Otherwise, skewed quantitative variables are expressed as medians [interquartile range (IQR)], and the Mann–Whitney test was used to analyze the differences. Univariate and multivariate logistic regression models were performed to identify potential predictors. All the variables included in Table 1 were first included in the univariate analysis to construct the model. Only variables that presented statistically significant differences (*p* < 0.05) in the univariate analysis and based on theoretical or empirical knowledge were considered to be related to the dependent variable and included in the multivariate analysis, as shown in Tables 2, 3. We measured the association between the model’s predictive variables and the dependent variable with the odds ratio (OR) and its corresponding 95% confidence interval (95% CI). Receiver operating characteristic (ROC) curves were generated, and the area under the curve (AUC) was calculated to summarize the predictive ability of potential predictors regarding MH. A *p* < 0.05 was regarded as statistically significant.

**TABLE 1:** Characteristics and clinical information for patients with Crohn’s disease.

	sMH (*n* = 17)	sNMH (*n* = 33)	*P*
Age of onset (y)	11.1 (3.2)	12.2 (2.0)	0.20
Male n (%)	12 (70.6)	21 (63.6)	0.76
Disease duration (m)	3.0 (14.7)	4.0 (8.5)	0.79
EEN duration (w)	9.0 (1.0)	9.0 (2.0)	0.54
Daily energy intake (kcal/d)	2213.0 ± 84.7	2238.0 ± 58.2	0.81
Weight gain (kg)	4.3 ± 0.5	5.7 ± 0.5	0.11
SES-CD at baseline	8.7 ± 1.2	16.2 ± 1.0	<0.001
SES-CD at the end of EEN	1.0 (3.5)	4.0 (2.0)	<0.01
Lewis score at baseline	902.0 (1657.5)	1104.0 (978.5)	0.93
Lewis score at the end of EEN	56.0 (269.5)	143.5 (333.0)	0.19
wPCDAI at baseline	37.1 ± 3.1	47.1 ± 2.9	0.04
wPCDAI at the end of EEN	3.8 (15.0)	7.5 (7.5)	0.94
FCP at baseline (μg/g)	1816.0 (583.0)	1900.0 (0.0)	0.08
FCP at the end of EEN (μg/g)	254.0 (113.0)	250.0 (656.0)	0.46
ESR at baseline (mm/h)	46.1 ± 9.1	67.9 ± 4.8	0.02
ESR at the end of EEN (mm/h)	14.0 (21.5)	11.0 (20.3)	0.89
Hgb at baseline (g/L)	120.6 ± 4.0	114.3 ± 3.2	0.24
Hgb at the end of EEN (g/L)	131.9 ± 3.2	128.1 ± 2.3	0.33
ALB at baseline (g/L)	40.2 ± 1.2	35.1 ± 1.1	<0.01
ALB at the end of EEN (g/L)	44.2 ± 0.7	44.2 ± 0.6	0.95
B2/B3 at baseline, n (%)	0 (0.0)	9 (27.3)	0.02
B2/B3 at ending, n (%)	2 (11.8)	5 (15.2)	1.00
B2/B3 at 1 year post-EEN, n (%)	0 (0.0)	6 (18.2)	0.08
P1 at baseline, n (%)	5 (29.4)	5 (15.2)	0.28
P1 at the end of EEN, n (%)	5 (29.4)	4 (12.1)	0.29
P1 at 1 year post-EEN, n (%)	2 (11.8)	3 (9.1)	1.00
AZA/MTX, n (%)	9 (52.9)	14 (42.4)	0.56
Infliximab, n (%)	7 (41.2)	27 (81.8)	0.01

*sMH, sustained mucosal healing; sNMH, sustained nonmucosal healing; y, year; m, month; w, week; EEN, exclusive enteral nutrition; SES-CD, Simple Endoscopic Score for Crohn’s Disease; wPCDAI, weighted Pediatric Crohn’s Disease Activity Index; FCP, fecal calprotectin; ESR erythrocyte sedimentation rate; ALB, albumin; Hgb, hemoglobin. AZA, azathioprine; MTX, methotrexate.*

**TABLE 2:** Predictive variables of MH at the end of EEN.

Variable	Univariate OR (95% CI)	*P*	Multivariate OR (95% CI)	*P*
SES-CD at baseline	1.44 (1.20–1.73)	0.00	1.40 (1.12–1.67)	0.00
ESR at baseline (mm/h)	1.02 (1.00–1.05)	0.04	/	/
ALB at baseline (g/L)	0.88 (0.79–0.98)	0.02	/	/
Hgb at baseline (g/L)	0.96 (0.93–1.00)	0.03	/	/
B2/B3	9.26 (1.06–80.93)	0.04	/	/

*dependent variable in multivariate analysis: MH at the end of EEN. n=50 patients. MH, mucosal healing; EEN, exclusive enteral nutrition; OR, odds ratio; 95% CI, 95% confidence interval; SES-CD, Simple Endoscopic Score for Crohn’s Disease; ESR erythrocyte sedimentation rate; ALB, albumin; Hgb, hemoglobin.*

**TABLE 3:** Predictive variables of sustained MH at 1-year post-EEN.

Variable	Univariate OR (95% CI)	*P*	Multivariate OR (95% CI)	*P*
SES-CD at baseline	1.29 (1.12–1.50)	0.001	1.33 (1.12–1.58)	0.001
wPCDAI at baseline	1.05 (1.00–1.10)	0.04	/	/
ESR at baseline (mm/h)	1.03 (1.00–1.05)	0.03	/	/
ALB at baseline (g/L)	0.85 (0.77–0.97)	0.01	/	/
**L(1,2,3) at baseline**				
L1		0.01	/	/
L2	40.00 (2.01–794.27)	0.02	/	/
L3	27.00 (2.92–249.48)	0.004	/	/
Early MH at the end of EEN	0.13 (0.04–0.51)	0.003	/	/

*dependent variable in multivariate analysis: MH at 1 year post.EEN. n = 50 patients. MH, mucosal healing; EEN, exclusive enteral nutrition; OR, odds ratio; 95% CI, 95% confidence interval; SES-CD, Simple Endoscopic Score for Crohn’s Disease; wPCDAI, weighted Pediatric Crohn’s Disease Activity Index; ESR, erythrocyte sedimentation rate; ALB, albumin.*

## Results

### Demographics and the Exclusive Enteral Nutrition Program

The demographic and clinical data of all patients in the two groups at baseline and at the end of EEN are summarized in Table 1. There were no significant age, sex, or disease duration differences between the two groups. There was also no difference in the EEN program (EEN duration, daily energy intake, and weight gain during the EEN process) between the two groups.

### Disease Activity

The SES-CD in the sMH group was lower than that in the sNMH group both at baseline and at the end of EEN [sMH vs. sNMH: 8.7 ± 1.2 vs. 16.2 ± 1.0, respectively, *p* < 0.001 at baseline; 1.0 (3.5) vs. 4.0 (2.0), respectively, *p* < 0.01 at the end of EEN] (Table 1). There were no significant differences in Lewis scores between the two groups at baseline or at the end of EEN (Table 1). The wPCDAI in the sMH group was lower than that in the sNMH group at baseline, but there was no difference at the end of EEN (sMH vs. sNMH: 37.1 ± 3.1 vs. 47.1 ± 2.9, respectively, *p* = 0.04 at baseline) (Table 1).

### Biochemical Markers

There were no significant differences in FCP or Hgb levels between the two groups at baseline or at the end of EEN (Table 1). The ESR in the sMH group was lower than that in the sNMH group at baseline, but there was no difference at the end of EEN (sMH vs. sNMH: 46.1 ± 9.1 vs. 67.9 ± 4.8, respectively, *p* = 0.02 at baseline) (Table 1). The ALB level in the sMH group was higher than that in the sNMH group at baseline, but there was no difference at the end of EEN (sMH vs. sNMH: 40.2 ± 1.2 vs. 35.1 ± 1.1, respectively, *p* < 0.01 at baseline) (Table 1).

### Paris Classifications at Each Time

**FIGURE 2:**
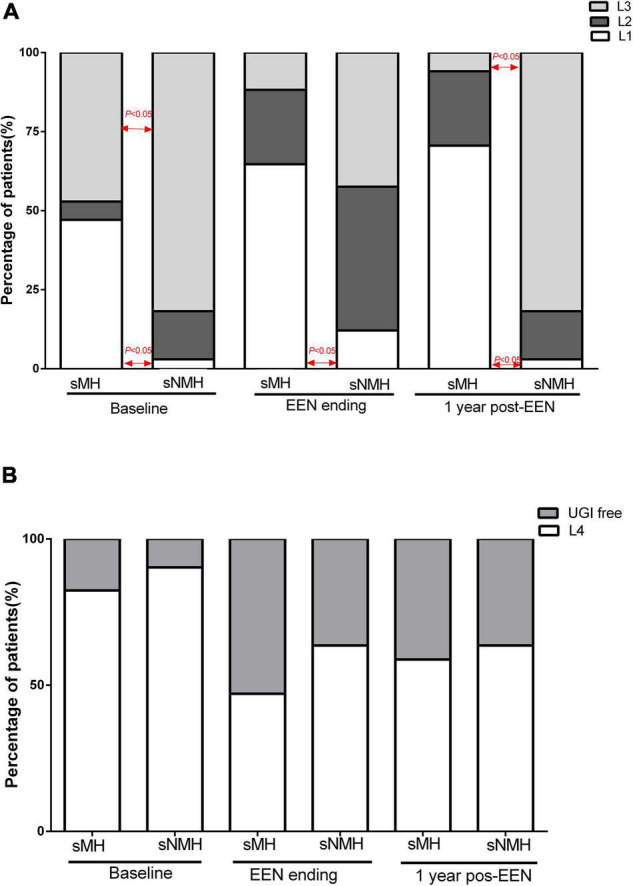
The Paris Classifications in the two groups of patients from baseline to 1-year post-EEN. **(A)** L1, L2, and L3 at each time point in the two groups of patients. **(B)** L4 at each time point in the two groups of patients. Abbreviations: EEN, exclusive enteral nutrition; sMH, sustained mucosal healing; sNMH, sustained non-mucosal healing; UGI, upper gastrointestinal.

From Figure 2A, we can see the variation trend of the Paris Classifications in the two groups of patients from baseline to 1-year post-EEN. In the sMH group, the number of patients classified with L1 or normal mucosa (at the end of EEN or 1-year post-EEN) increased during follow-up, so there were more patients classified with L1 or normal mucosa in the sMH group at each time point [sMH vs. sNMH: 8 (47.1%) vs. 1 (3.0%), respectively, *p* < 0.001 at baseline; 11 (64.7%) vs. 4 (12.1%), respectively, *p* < 0.001 at the end of EEN; 12 (70.6%) vs. 1 (3.0%), respectively, *p* < 0.001 at 1-year post-EEN]. In the sMH group, the number of patients classified with L3 decreased during follow-up. However, in the sNMH group, patients classified with L3 decreased at the end of EEN but increased again at 1-year post-EEN during follow up, so there were fewer patients classified with L3 in the sMH group at baseline and at 1-year post-EEN, but no difference was shown at the end of EEN compared with the sNMH group [sMH vs. sNMH: 8 (47.1%) vs. 27 (81.8%), respectively, *p* = 0.02 at baseline; 2 (11.8%) vs. 14 (42.4%), respectively, *p* = 0.05 at the end of EEN; 1 (5.9%) vs. 27 (81.8%), respectively, *p* < 0.001 at 1-year post-EEN]. There were no differences in patients classified with L2 (Figure 2A), L4 (Figure 2B), and P1 (Table 1) between the two groups at each time point. At baseline, fewer patients were classified with B2/B3 in the sMH group [sMH vs. sNMH: 0 (0.0%) vs. 9 (7.3%), respectively, *p* = 0.02 at baseline], but at the end of EEN and at 1-year post-EEN, there were no differences in B2/B3 classification between the two groups (Table 1).

### Maintenance Treatment After Exclusive Enteral Nutrition

**FIGURE 3:**
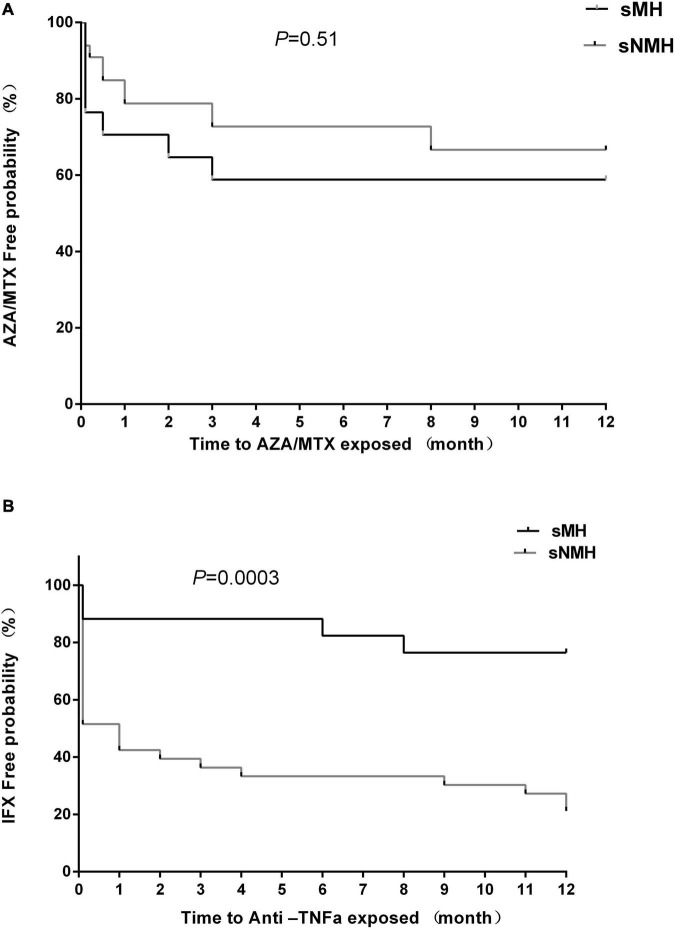
Kaplan–Meier curve depicting the difference in the AZA/MTX-free **(A)** and infliximab-free **(B)** probability between the sMH and sNMH groups after EEN induction. Abbreviations: sMH, sustained mucosal healing; sNMH, sustained non-mucosal healing; AZA, azathioprine; MTX, methotrexate; IFX, infliximab.

For azathioprine (AZA)/MTX usage, there were no differences in the percentage of patients (Table 1) or time to exposure (Figure 3A) between the two groups. For infliximab (IFX), fewer patients received IFX treatment in the sMH group than in the sNMH group [7 (41.2%) vs. 27 (81.8%), respectively, *p* = 0.01] (Table 1), and there was a longer exposure time to IFX in the sMH group than in the sNMH group (Figure 3B, *p* < 0.001).

### The Predictor of Mucosal Healing to Exclusive Enteral Nutrition Treatment

#### Short-Term: At the End of Exclusive Enteral Nutrition

Among these 50 patients, 23 patients had early MH at the end of EEN, so we first tried to identify variables at baseline that were associated with early MH at the end of EEN, as shown in Table 1. We applied a two-step strategy based on a logistic regression model. In univariate logistic regression analysis, the SES-CD, ESR, ALB level, Hgb level, and B2/B3 classification at baseline were significantly associated with MH at the end of EEN (Table 2). Multivariate logistic regression analysis was then performed to assess these independent factors. Only the SES-CD at baseline was negatively associated with MH at the end of EEN (OR = 1.40 95% CI = 1.12–1.67, *p* = 0.00 in the multivariate model) (Table 2).

#### Long Term: 1-Year Post-EEN

To identify the variables associated with MH at 1-year post-EEN, we also applied a two-step strategy based on a logistic regression model, as shown in Table 1. In univariate logistic regression analysis, the SES-CD, wPCDAI, ESR, ALB level, L (1,2,3) classification at baseline, and early MH at the end of EEN were significantly associated with MH at 1-year post-EEN (Table 3). However, in the multivariate logistic regression model, only the SES-CD at baseline was negatively associated with MH at 1-year post-EEN (OR = 1.33, 95% CI = 1.12–1.58, *p* = 0.001 in the multivariate model), and other variables were finally not included in the multivariate analysis (Table 3).

#### The Optimal Cutoff Value of the SES-CD at Baseline

**FIGURE 4:**
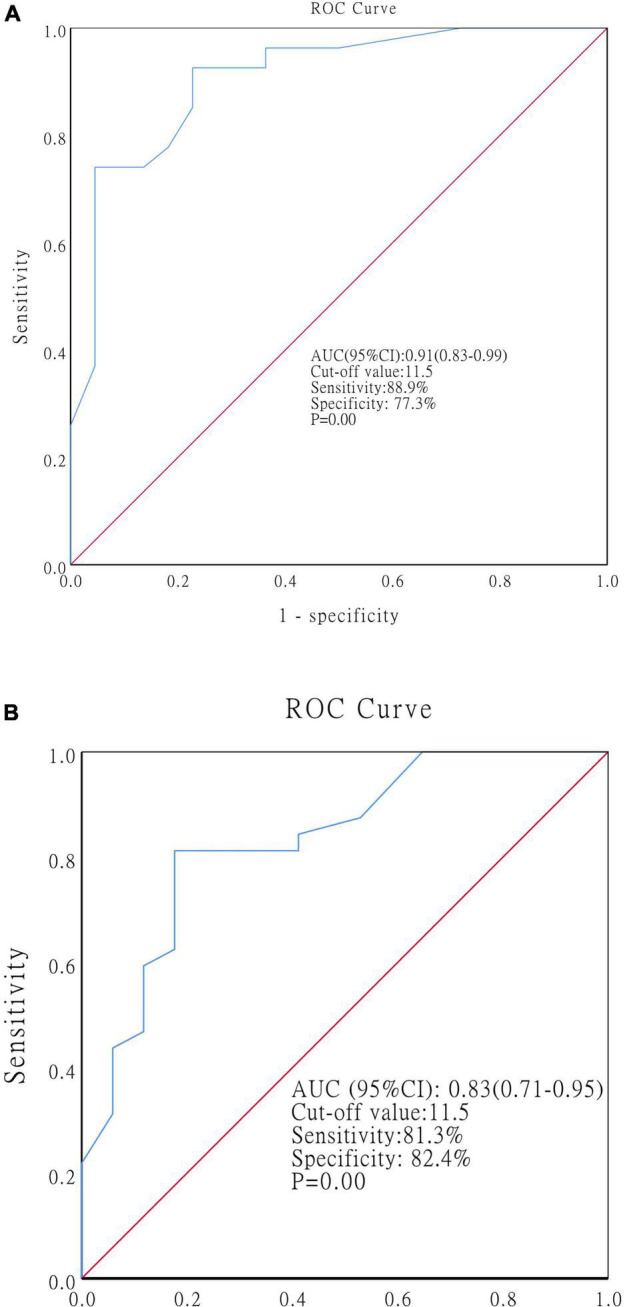
ROC curves showing the optimal cutoff value of the SES-CD at baseline for predicting MH at the end of EEN **(A)** and at 1-year post-EEN **(B)**. Abbreviations: ROC, receiver operating characteristics; SES-CD, simple endoscopic score for CD; MH, mucosal healing; EEN, exclusive enteral nutrition.

Receiver operating characteristic curves were generated to determine the optimal cutoff value of the SES-CD at baseline for predicting MH at the end of EEN and 1-year post-EEN. At the end of EEN, the SES-CD at baseline showed an AUC of 0.91 (cutoff value: 11.5, sensitivity: 88.9%, specificity: 77.3%, *p* = 0.00, 95% CI: 0.83–0.99) (Figure 4A). At 1-year post-EEN, the SES-CD at baseline showed an AUC of 0.83 (cutoff value: 11.5, sensitivity: 81.3%, specificity: 82.4%, *p* = 0.00, 95% CI: 0.71–0.95) (Figure 4B).

### Two-Year Follow-Up Post-EEN

Nine patients in the sMH group and 15 in the sNMH group completed the 2-year follow-up. Among them, eight patients maintained MH in the sMH group, and only three patients reached MH in the sNMH group [sMH vs. sNMH: 8 (88.9%) vs. 3 (20.0%), respectively, *p* = 0.002]. Two patients received IFX in the sMH group, and 13 patients received anti-TNF therapy (IFX or adalimumab) [sMH vs. sNMH: 2 (22.2%) vs. 13 (86.7%), respectively, *p* = 0.003]. No patients in the sMH group needed surgery, but two patients in the sNMH group received surgery therapy.

## Discussion

Very few studies have examined long-term outcomes after EEN. In this study, we evaluated MH in pediatric CD patients 1-year post-EEN and found that the SES-CD at baseline could predict both short- and long-term MH after EEN.

In this study, the early MH rate at the end of EEN was 46%, similar to that in previous studies (11–15). One year after EEN, the MH rate in our patients was 34.0%, patients in the sMH group relapsed, and most patients in the sNMH group maintained NMH after EEN. MH is difficult for CD patients to achieve, not to mention the difficulty in maintaining sustained MH (20). It is better to find the factors associated with MH after EEN in the short- and long-term to improve the disease process of CD patients. For the EEN protocol, Table 1 shows that all patients in the two groups received high-energy intake each day, had an appropriate EEN duration (the median was 9 weeks in the 2 groups), and achieved excellent nutrient improvement (weight gain and Hgb and ALB levels), and clinical (wPCDAI), biochemical (ESR), and endoscopic responses during the EEN process, indicating that our EEN protocol was appropriate. More patients and a shorter exposure time to anti-TNFα were found in the sNMH group after EEN, meaning the maintenance choice was more aggressive in the sNMH group after EEN.

We found differences in the SES-CD, wPCDAI, ESR, and ALB levels at baseline between the two groups. Grover et al. (14) compared patients with sustained remission with patients with relapse 1-year after EEN, and they did not find that the variables pre-EEN, including the SES-CD, were different between the two groups. These results were different from our results and may need more similar studies in the future. However, studies have demonstrated that a wPCDAI < 57 and an FCP level < 500 μg/g could be predictive factors of the response to EEN (18). We did not find that the wPCDAI, and any biochemical markers, including the ESR, ALB level, and FCP level, were associated with MH after EEN in the multivariate model.

Several studies have shown that newly diagnosed pediatric CD patients who failed to reach clinical remission after induction therapy had predictors of poor outcomes (21–23). Early endoscopic remission has been reported to improve outcomes at the 1-year follow-up (14). This could also be found in our patients. In this study, among 23 patients who reached early MH at the end of EEN, 13 maintained sustained MH at 1-year post-EEN under immunomodulatory treatment. Among the 27 patients who did not reach early MH at the end of EEN, only 4 patients reached MH 1-year post-EEN. Poorer outcomes in the sNMH group were also observed 2-year post-EEN. However, early MH at the end of EEN was not associated with 1-year post-EEN in the multivariate logistic regression model, perhaps because of the high relapse rate in CD patients.

The SES-CD is the primary tool for measuring mucosal inflammation in clinical practice. Previous studies found that the SES-CD was strongly associated with the risk of surgery (24) and clinical recurrence in CD patients (25), and a low SES-CD at baseline was associated with the response to ustekinumab (26). For EEN, a study demonstrated that the SES-CD was related to the clinical response to EEN in colonic CD patients (27). In this study, we found that the SES-CD at baseline was associated with MH both at the end of EEN and 1-year post-EEN; to the best of our knowledge, this has not been reported before. We calculated that the cutoff value was 11.5. To obtain early MH and maintain sustained MH, patients with an SES-CD greater than 11.5 at baseline should be treated more aggressively with biologics. Because some more severe patients could combine IFX with EEN to induce remission, it has been reported that the combination of EEN and TNFα inhibitors was associated with faster clinical remission (5).

Whether the location of the disease influences the outcome of EEN is unclear (28). However, a study demonstrated that patients with isolated colonic classification (L2) showed a lower response to EEN treatment than patients with other classifications (29, 30), and ileal involvement could be a predictive factor of the response to EEN (18). We found more patients with L1 classification in the sMH group at each time point from baseline to 1-year post-EEN and fewer patients with L3 and B2/B3 classification in the sMH group at baseline than in the sNMH group. From Table 3, we can see that compared with the patients who presented with L1 classification, the patients with L2 or L3 classification had a high risk of maintaining NMH at 1-year post-EEN. However, we did not find an association of Paris Classifications with MH after EEN in the multivariate mode because of the limited sample size.

In conclusion, we found that the SES-CD at baseline was associated with MH at the end of EEN and 1-year post-EEN. Patients with an SES-CD < 11.5 had a high probability of reaching MH by EEN-inducing therapy and maintained sustained MH at 1-year post-EEN under immunomodulation. We suggest that patients with an SES-CD greater than 11.5 at baseline should be treated more aggressively with biologics.

This study has some limitations. This was a single-center study with a small sample size, which may cause us to not find an association of Paris Classifications with MH after EEN. In the future, we need to obtain more reliable and representative data to find the association of Paris Classifications with MH after EEN.

## Data Availability Statement

The original contributions presented in the study are included in the article/Supplementary Material, further inquiries can be directed to the corresponding authors.

## Ethics Statement

The studies involving human participants were reviewed and approved by Ethics Committee of the Children’s Hospital of Fudan University ([2017]131). Written informed consent to participate in this study was provided by the participants’ legal guardian/next of kin.

## Author Contributions

YH, YW, and WT contributed to the design of the study and interpretation of data. WT, WH, and PS performed the statistical analysis. WT, ZY, JW, and YZ performed the acquisition of data. WT drafted the manuscript. YH critically revised the manuscript for important intellectual content. All authors reviewed and approved the final version of the manuscript.

## Conflict of Interest

The authors declare that the research was conducted in the absence of any commercial or financial relationships that could be construed as a potential conflict of interest.

## Publisher’s Note

All claims expressed in this article are solely those of the authors and do not necessarily represent those of their affiliated organizations, or those of the publisher, the editors and the reviewers. Any product that may be evaluated in this article, or claim that may be made by its manufacturer, is not guaranteed or endorsed by the publisher.
