# Supplementary material for: The SES-CD Could Be a Predictor of Short- and Long-Term Mucosal Healing After Exclusive Enteral Nutrition in Pediatric Crohn’s Disease Patients
Source: Front Pediatr. 2022 May 18;10:874425. doi: 10.3389/fped.2022.874425 (PMC9157786; doi:10.3389/fped.2022.874425)
Supplement: Supplementary file 1 [file Table_1.DOCX]

Supplementary Table1 the recommended energy intake for the patients

enrolled in this study

|  | Male | Female |
| --- | --- | --- |
| 1-3 years | 110kcal/kg/d | 110 kcal/kg/d |
| 4-6 years | 100kcal/kg/d | 100 kcal/kg/d |
| 7-10 years | 2170 kcal/d | 1945kcal/d |
| 11-14 years | 2300 kcal/d | 2045 kcal/d |
| 15-18 years | 2755 kcal/d | 2300 kcal/d |
